# Mothers' Experiences of Formula Feeding Support in the UK: A Qualitative Systematic Review

**DOI:** 10.1111/mcn.70182

**Published:** 2026-04-17

**Authors:** Eilish James, Gary Hoang, Daniel Lange, Kate Jolly, Joanne Clarke

**Affiliations:** ^1^ Department of Applied Health Sciences, School of Health Sciences, College of Medicine and Health University of Birmingham Birmingham UK; ^2^ Sandwell Metropolitan Borough Council Oldbury UK; ^3^ College of Medicine and Health University of Birmingham Birmingham UK

**Keywords:** bottle feeding, formula feeding, infant feeding, infant formula, qualitative, support, systematic review

## Abstract

Most babies in the UK and Ireland receive formula milk in their first 6 months of life. Understanding mothers' perceptions of formula feeding support is crucial in shaping practical guidelines, research‐based strategies and future policies to support formula feeding decisions. This review aimed to synthesise qualitative evidence on mothers' experiences of formula feeding support in the UK and Ireland. The Cochrane Handbook guidance for systematic reviews was followed and MEDLINE, CINAHL, PsycINFO, Web of Science and ASSIA (ProQuest) databases were searched. Ten included papers were assessed for methodological quality using a CASP checklist. The Thomas and Harden three‐stage approach was used to thematically synthesise the data. The synthesised findings include: (1) Limited support for formula feeding, (2) Withheld or conflicting healthcare professional support, and (3) Emotional health and wellbeing impact on mothers. Mothers who formula feed require empathetic, formal guidance from HCPs as they navigate their feeding journey. This review highlights predominantly negative experiences due to inadequate support received. Formula feeding support should be recognised as an essential element in perinatal care. Future research could investigate effective interventions for formula feeding support strategies.

## Summary

- HCPs should provide tailored, non‐judgemental support for all infant feeding methods, including formula feeding, to build maternal confidence and reduce stigma.
- HCPs should provide clear, consistent, evidence‐based feeding advice to reduce mothers' reliance on informal sources of formula milk marketing.
- Further research should be conducted to understand HCPs' perspectives on providing formula feeding support, their awareness of guidance and how to operationalise it.

## 1 Introduction

Breastfeeding has well‐established benefits for infants and mothers (Victora et al. 2016), with the World Health Organization recommending exclusive breastfeeding for 6 months (UNICEF 2023). Despite this, average duration of breastfeeding in the UK remains low, with a rapid drop‐off in the weeks following birth. Any breastfeeding at 6–8 weeks ranges from 31.6% to 52.7% in the UK devolved countries (Public Health Agency 2021; Public Health England 2021; Public Health Scotland 2021; Welsh Government 2021), with only 1% of infants exclusively breastfed up to 6 months (McAndrew et al. 2012). Breastfeeding rates in the Republic of Ireland are similarly low; 35% of mothers report any breastfeeding at 3 months (Theurich et al. 2019). As formula milk is the only alternative to breastmilk for infants, these data suggest that most babies in the UK and Ireland receive formula milk in their first 6 months.

Socio‐economic disparities in infant feeding practices in the UK contribute to health inequality, with mothers who are young, white, from disadvantaged backgrounds, who have lower educational qualifications, or were formula fed themselves, being more likely to formula feed (Public Health Agency 2021; Public Health England 2021; Public Health Scotland 2021; Welsh Government 2021). Although conducted 15 years ago, the most recent UK Infant Feeding Survey (McAndrew et al. 2012) found that almost half of mothers who prepared powdered infant formula did not follow recommendations intended to reduce the risk of infection and overconcentration of feeds, such as adding water to a bottle before powder and correct temperature of the water. These sub‐optimal preparation practices are associated with greater risk of illness and mortality (World Health Organisation 2012). Evidence also links excess weight and obesity to poor formula feeding practice, with over‐feeding and rapid weight gain during infancy shown to predispose to later obesity (Appleton et al. 2018; Woo Baidal et al. 2016).

There are some circumstances in which women are advised not to breastfeed, such as HIV‐positive status (British HIV Association 2020). Mothers choose formula feeding for various reasons, and evidence suggests this is not due to a knowledge gap (Dattilo et al. 2020). Qualitative data from a UK survey of 624 formula feeding mothers to explore reasons for formula feeding identified four themes: feeding problems, mental health, feeding attitudes and sharing the load, with negative attitudes towards breastfeeding being especially important for mothers who had never breastfed (Roberts et al. 2023). A scoping review of the reasons why mothers decide to cease exclusive breastfeeding within the first 6 months found that lactation issues were the most common reason for cessation (Olalere and Harley 2024). A systematic review identified three main reasons for mixed feeding: perceived necessity (concerns about infant health and breastfeeding difficulties), perceived external influences (returning to work and socio‐cultural attitudes), and perceived choice (convenience, flexibility, and co‐parent involvement) (Monge‐Montero et al. 2024). Additionally, Hauck et al. (2021) highlighted challenges such as breastfeeding in public and the lack of safe spaces.

In this review, we conceptualise support as emotional, informational, and practical assistance (House 1983). In an infant feeding context, support can be provided by family, friends, healthcare professionals (HCPs) and community sources, with a wide range of acts of support important to mothers (Myers et al. 2021). The UNICEF UK Baby Friendly Initiative (BFI), introduced in 1992, highlights the importance of support for parents who formula feed, focusing on fostering closeness, sharing information about infant formula preparation and selection, shielding families from commercial pressures, and advocating for all infants (UNICEF 2016). The Royal College of Nursing (RCN) guidance aligns with BFI (Royal College of Nursing 2016). Similarly, the National Institute for Health and Care Excellence (NICE) postnatal care guidance emphasises the importance of HCPs providing advice and information to help parents make informed decisions with interventions including assistance for those who formula feed (National Institute for Health and Care Excellence 2021).

To improve support provided to mothers, it is important to understand their experiences of support for formula feeding their infants. A systematic review of mothers' experiences of formula feeding identified that women were uncertain about how to formula feed and reported not getting enough information from health professionals (Lakshman et al. 2009). A new review, focusing on experiences of support for formula feeding would identify whether women's experiences have changed in the intervening period in a post‐BFI context. This review examines mothers' experiences of support for formula feeding in the UK and Ireland and explores potential approaches to enhancing the provision of support to promote informed and safe formula feeding practices.

## 2 Methods

The review was guided by the Cochrane Handbook for systematic reviews (Chandler et al. 2019) and written according to the Preferred Reporting Items of Systematic Reviews and Meta‐Analysis checklist (PRISMA) (Shamseer et al. 2015). The Enhancing Transparency in Reporting the synthesis of Qualitative research checklist (ENTREQ) (Tong et al. 2012) was used to guide the reporting standards and project outputs. The protocol was registered on PROSPERO (CRD42024555511).

### 2.1 Eligibility Criteria

Included studies focused on the views and experiences of mothers in the UK or Ireland on the support for formula feeding their baby up to the age of 12 months. Support could be from any source, in any setting. Studies were included on experiences of support for formula feeding where the mother was solely formula feeding or mixed feeding. Studies focusing on mothers' experiences of breastfeeding support, and studies conducted outside of the UK or Ireland were excluded.

The review included relevant peer‐reviewed studies published in English from 1992 (to reflect the establishment of the UNICEF UK Baby Friendly Initiative (BFI) (UNICEF 2016)) to June 2025. Qualitative or mixed methods studies using focus groups or interviews were included. To include qualitative data with the most contextual depth and richness, data from open‐ended survey questions were excluded (Flemming and Noyes 2021). All qualitative theoretical approaches and data synthesis methods were included. Quantitative studies and studies not peer‐reviewed were excluded.

A SPIDER framework (Cooke et al. 2012) was used to define search terms, with ‘phenomenon of interest’ duplicated to accommodate search terms for both ‘formula feeding’ and ‘support’ (Table 1).

**Table 1:** SPIDER framework applied to search terms.

Sample population	Phenomenon of interest (a)	Phenomenon of interest (b)	Design	Evaluation	Research type
Mother* England Scotland Wales Northern Ireland Ireland	Formula*‐feed* Bottle feed* Infant feed* Infant formula Formula milk	Support Advice Guidance Assistance Education Peer support Intervention Midwife Health visitor GP Health professional Doctor Nurse Family Friends Online Social Media	Qualitative Interview Focus groups	Experience* Perception* View* Attitude* Opinion* Satisfaction Emotion Feeling	Qualitative Mixed methods

* Truncation symbol used to search databases for word ending variants.

### 2.2 Search Strategy

A combination of keyword searches and medical subject headings (MeSH) terms, using Boolean operators “AND” and “OR” were used to conduct the search in MEDLINE, CINAHL, PsycINFO, Web of Science, and ASSIA (ProQuest). The search strategy for all databases is available in Supporting Information file 1. Searches were conducted in May 2024 and re‐run on 12th June 2025. Reference lists of included papers were hand‐searched.

### 2.3 Study Selection and Quality Assessment

Titles and abstracts were imported into Covidence for screening and firstly assessed by EJ or GH. Full‐text copies of papers meeting the inclusion criteria, and those where inclusion was uncertain based on title and abstract were retrieved and reviewed against inclusion criteria. A second reviewer (DL or JC) sampled a minimum of 50% of studies at title and abstract screening stage, and a minimum of 10% of studies at full text review stage. Where reviewers disagreed, this was overcome through discussion, and it was not necessary to involve a third reviewer.

Following the full text review stage, the decision was made to exclude two papers that focused on the experiences of HIV positive mothers (Tariq et al. 2016; Rai et al. 2023). This population has a unique reason to formula feed and specific guidelines to recommend formula feeding (British HIV Association 2020); therefore, it was felt that this population should not be analysed alongside the UK general population.

All included studies were assessed using the CASP qualitative research appraisal tool (Critical Appraisal Skills Programme 2024) by two reviewers (EJ and JC). Consensus for any discrepancies was reached by discussion. Studies were not excluded based on CASP assessment. As all studies were found to be of good methodological quality, the appraisal process provided assurance of the robustness of the evidence base. Therefore, all studies contributed equally to the synthesis and quality was not a consideration in our interpretation of the results.

Inter‐reviewer agreement statistics were not calculated; discrepancies between reviewers were addressed through discussion and consensus rather than quantified agreement.

### 2.4 Data Extraction

A data extraction form was used to extract basic study information (author, year, title), study characteristics (country, interview setting, population including demographic information, recruitment method, sample number, data collection date, data collection method, analysis method), study aims, study outputs (main themes, data from the results, discussion and conclusion section, author recommendations) and limitations identified through CASP implementation. Data were defined as first order (participants' direct quotes) and second order constructs (author interpretation, statements, assumptions and ideas), within the results, discussion and conclusion sections. Data were extracted where they contributed to answering the review question.

### 2.5 Data Synthesis

The Thomas and Harden (2008) three‐stage approach to thematic synthesis was used. Extracted outcome data were imported into NVivo (v12) for stage one ‐ open code generation. Line‐by‐line code generation was employed, to capture mothers' experiences of formula feeding support. Throughout this stage, the coding process was iterative, involving constant comparison between codes and the data to ensure that each code accurately represented participants' views. New codes were developed as new ideas were identified, and existing codes were collapsed or refined to better capture nuances of the data. Some of the codes became descriptive themes themselves. The second stage of generating descriptive themes was completed by organising similar or related codes from across the studies and labelled. The third stage of generating analytical themes was developed by interpreting stage two and considering the review question through an on‐going process of refinement. All stages were completed independently by one reviewer (EJ) with the second reviewer (DL) reviewing and verifying the analytical themes to ensure the findings appropriately reflected the original data, through a series of meetings. All authors agreed the final synthesised findings.

### 2.6 Reflexivity Statement

EJ undertook the systematic review as part of a Master's in Public Health, supervised by JC and KJ, both of whom are academics with qualitative research experience in the field of infant feeding and public health. GH and DL are male medical student and public health registrar respectively, who contributed to literature searching and study selection, with DL also involved in data extraction and discussions around thematic analysis. EJ, JC and KJ have personal experience as mothers who have mixed‐fed babies and understand the role this experience could have played in introducing bias given the interpretative nature of thematic synthesis. Following PRISMA guidelines and the application of predetermined criteria for study selection and data extraction, as well as frequent discussions between authors helped ensure a rigorous approach to the thematic synthesis.

## 3 Findings

### 3.1 Study Selection

**Figure 1:**
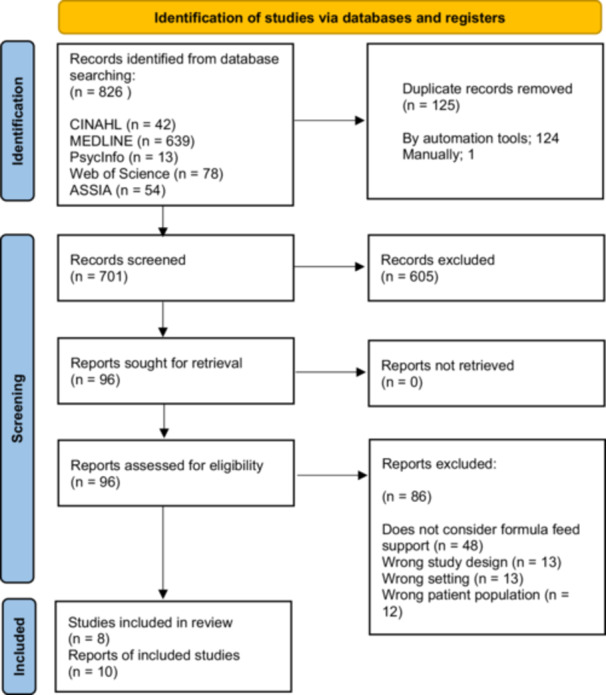
PRISMA flow chart of study selection.

Database searches yielded 826 results; no additional papers were identified through reference checking of included papers. We removed 125 duplicates and excluded 605 records through title and abstract screening. Of the 96 full‐text papers accessed, 10 papers, relating to 8 datasets, were eligible for data extraction and analysis. Two studies each produced two papers answering different research questions—Hoddinott and Pill (1999) and Hoddinott and Pill (2000); Lee (2007a) and Lee (2007b). All included studies were published in English. The full selection process is outlined in a PRISMA flowchart (Figure 1).

### 3.2 Characteristics of Included Studies

Study characteristics are presented in Table 2. All studies were conducted in the UK with eight from England, two from Scotland, and two across the UK; none were from Ireland.

**Table 2:** Included studies characteristics summary.

Study, location	Study aim	Participants	Data collection method	Data analysis	Key findings
Conway et al. (2023) England, Scotland, Wales	To understand how mothers use commercial milk formula (CMF) labels to inform their feeding choices and explore mothers' understanding of differences between CMF products.	25 mothers using CMF for children aged < 3 years old.	Semi‐structured interviews	Thematic analysis	Three main themes: (1) It's all about the brand, (2) formula stages exist for a reason, (3) presentation matters. Limited support received from HCPs caused mothers to seek advice from alternative sources, such as friends, family members and websites.
Guell et al. (2018) England	To explore women's experiences of participating in a trial to reduce infant formula milk intake and promote healthy weight gain.	19 formula feeding mothers with infants age 7 months old on average.	Semi‐structured interviews	Thematic analysis	Three main themes: (1) Receiving professional attention when support was felt withheld, (2) Receiving trial advice on the backdrop of disparate, informal, and often conflicting information, (3) Joining the trial with an older infant demanded too much change of entrenched practices.
Hoddinott and Pill (1999) England*	To examine antenatal expectations and postnatal experiences of first‐time mothers to provide an insight into postnatal discontent and why so many women change their feeding behaviour in the first few weeks after birth.	21 first‐time mothers, London.	Semi‐structured interviews	Framework, discourse, grounded	Mothers with prior baby care experience felt confident formula feeding, rarely seeking out professional help. Formula‐feeding mothers highlighted inadequate support received from professionals. Majority of mothers described barriers in accessing help from HCPs.
Hoddinott and Pill (2000) England*	To look at how communication by HCPs about infant feeding is perceived by first‐time mothers.	21 first‐time mothers, London.	Semi‐structured interviews	Framework, discourse, grounded	Mothers who formula‐fed experienced gaps in care and information received in comparison to mothers choosing to breastfeed on postnatal wards.
Lagan et al. (2014) Scotland	To explore the expectations and experiences of postnatal mothers in relation to infant feeding, and to identify how care could be improved.	78 mothers with any infant feeding intention.	Semi‐structured interviews and focus groups	Framework approach	Participants expressed strong perceptions that midwives could not provide support for those formula‐feeding and reported feelings of pressure to breastfeed.
Lakshman et al. (2011) England	To explore the views of HCPs and formula‐feeding mothers on: (1) the Programme for Healthy Growth and Nutrition during infancy; (2) the trial design for the planned Baby Milk trial (3) two draft leaflets (to assess whether they were useful, easy to understand, relevant and acceptable).	11 formula‐feeding mothers.	Semi‐structured interviews and focus groups	Thematic analysis	Mothers expressed a lack of a support due to conflicting advice from HCPs and other sources. The programme's intervention provided non‐judgemental support validating mother's choices to formula feed. Mothers emphasised the need for empathetic support in a non‐judgemental, consistent manner.
Lee (2007a) England*	To explore the experiences of women who formula‐fed their babies during the first three months of their child's life.	33 formula‐feeding mothers when infant 0–3 months old.	Semi‐structured interviews	Qualitative analysis	Some women did not like the ‘breast is best’ message and reported feeling guilty and hiding the fact that they formula fed their babies from HCPs. Women reported a lack of support for formula feeding and reported feeling lonely, worried and bewildered.
Lee (2007b) England*	To explore how the pro‐breastfeeding message as an aspect of morality, influences maternal identity.	33 formula‐feeding mothers when infant 0–3 months old.	Semi‐structured interviews	Qualitative analysis	Women found it difficult to find information and support about formula feeding from HCPs. Women who had planned to breastfeed found it especially difficult as they were unprepared.
Symon et al. (2012) Scotland	To examine whether antenatal feeding intention was associated with satisfaction with infant feeding method; and to explore similarities and differences in infant feeding experience of women with different antenatal feeding intentions.	292 women over 30 weeks' gestation; 60 had no intention to breastfeed, 130 were undecided and 100 had high intentions to breastfeed.	Semi‐structured interviews	Thematic analysis	Half of women with ‘no intention to breastfeed’ reported professional support was available but generally unhelpful. Some mothers experienced pressure to breastfeed from HCPs and family members. Mixed family support received.
Thomson et al. (2015) England	To explore mothers' experiences, opinions and perceptions of infant feeding.	63 mothers	Semi‐structured interviews and focus groups	Framework analysis	Three main themes: (1) exposure of women's bodies and infant feeding methods, (2) undermining and inadequate support, (3) perceptions of inadequate mothering.

*These studies used the same population sample.

Abbreviation: HCP, healthcare professional.

A total of 302 participants were included within this review, accounting for the two papers that report data from the same sample population, and for inclusion only of the study participants meeting the inclusion criteria. The number of participants in the included studies ranged from 11 to 292. (Table 2).

Study participants were a mixture of primiparous and multiparous mothers with infants under 12 months old. There was a mixture of exclusive formula‐feeders and mixed‐feeders across studies, and distinction between the two groups could not be made within this review. Most of the study population from the included studies were white and from a range of socioeconomic backgrounds and ages.

### 3.3 Quality Assessment

Overall, quality of studies was good. Most papers provided a clear statement of aims; all used an appropriate qualitative methodology and adopted suitable research designs. Strategies for participant recruitment were generally appropriate and data collection methods were well aligned with research objectives. Ethical issues were considered, and findings were clearly presented and valuable across all studies. The main area of weakness was a lack of consideration of the relationship between researchers and participants. Table 3 shows a summary of quality assessment results.

**Table 3:** Quality assessment summary of the included studies.

Study	Clear aims	Methodology appropriate	Research design appropriate	Recruitment strategy appropriate	Data collection appropriate	Relationship adequately considered	Ethical issues considered	Data analysis rigorous	Clear statement of findings	Research valuable
Conway et al. (2023)	Y	Y	Y	Y	Y	Y	Y	Y	Y	Y
Guell et al. (2018)	Y	Y	Y	Y	Y	Y	Y	Y	Y	Y
Hoddinott and Pill (1999)	Y	Y	Y	Y	Y	Y	Y	Y	Y	Y
Hoddinott and Pill (2000)	Y	Y	Y	Y	Y	Y	Y	Y	Y	Y
Lagan et al. (2014)	Y	Y	Y	Y	Y	N	Y	Y	Y	Y
Lakshman et al. (2011)	Y	Y	Y	N	Y	N	Y	Y	Y	Y
Lee (2007a)	Y	Y	Y	Y	Y	N	Y	U	Y	Y
Lee (2007b)	U	Y	Y	Y	Y	N	Y	U	Y	Y
Symon et al. 2012	Y	Y	Y	Y	Y	N	Y	Y	Y	Y
Thomson et al. (2015)	Y	Y	Y	Y	Y	N	Y	Y	Y	Y

Abbreviations: N, no; U, unclear; Y, yes.

### 3.4 Thematic Synthesis

From the data synthesis, nine descriptive themes were identified under three analytical themes: ‘Limited support for formula feeding’, ‘Withheld or conflicting healthcare professional support’, and ‘Emotional health and wellbeing impact on mothers’ (Table 4). Themes are presented with illustrated quotes.

**Table 4:** Thematic summaries.

Analytical themes	Descriptive themes
1 ‐ Limited support for formula feeding	1a ‐ Sources of formula feeding support
	1b ‐ Dissatisfaction in formula feeding support
	1c ‐ Feeling unprepared for motherhood
	1 d ‐ Marketing as a source of information
	1e ‐ Positive experiences of personalised, formal support
2 ‐ Withheld or conflicting healthcare professional support	2a ‐ Perception of healthcare professionals withholding support
	2b ‐ Conflict in healthcare professional support
3 ‐ Emotional health and wellbeing impact on mothers	3a ‐ Experiences of stigma in infant feeding decisions
	3b ‐ Postpartum vulnerability

### 3.5 Themes

#### 3.5.1 Limited Support for Formula Feeding

Mothers reported receiving support for formula feeding from friends and family ‘*My mum showed me how to make the bottles’*. (Hoddinott and Pill 1999, p.560) and HCPs ‘*The midwife came up to show me ‐ about 24 h later’*. (Hoddinott and Pill 2000, p. 229). However, the support was commonly seen as limited or delayed and evidence across all studies highlighted negative experiences as a common theme among mothers. Widespread dissatisfaction was consistently noted when mothers discussed their formula feeding support experiences, or the lack thereof. Women often felt that formula feeding was unsupported within healthcare and they had to figure it out alone.

Not really any support with bottle feeding, this is my second child and first time there was none really either, just have to find out for yourself really.

(Symon et al. 2013, p. e51).

A perceived lack of support left women feeling lacking in confidence and inadequately prepared for motherhood with one woman reflecting that feeding her baby ‘…*could have been a more enjoyable experience for the baby and for me if we'd known some of the pitfalls to look for upfront*’. (Lagan et al. 2014, p.e52)

There was uncertainty on formula milk selection, switching between brands, safe preparation of bottles, determining milk quantities and deciding whether follow‐on‐formula was necessary. These gaps in information led women to look for alternative sources of support. Some women reported anecdotal evidence, online resources and reading materials to support feeding decisions; others reported ‘*It was bewildering really’* (Lee 2007b, p. 1084) and having no information, leading to a trial‐and‐error approach:

My husband went out to buy formula, but he didn't know what to buy. We didn't have any information, so he bought one of each kind. We thought we'd try each and see which one he likes best, but of course, that's not the way you do it.

(Lee 2007a, p. 305)

With a lack of professional support, some women turned to marketing and advertising of formula milk as a source of information: ‘*It's actually literally just been from advertising, sort of, I guess a trusted brand name that you've kind of heard, especially being a new mum, you want something you know’.* (Conway et al. 2023, p. 1011). This reliance on information from advertising may have contributed to perceptions of formula feeding as a risky practice:

Everything says ‘breast is best.’ Even on the formula cans, ‘This is not a substitute for breastfeeding.’ Everything comes with a health warning. You almost feel like you are feeding your baby poison. But it's not! It's milk. Just milk.

(Lee 2007a p. 295)

Positive experiences of formula feeding support were uncommon and reported when mothers participated in formula feeding support trials where they received ‘…*loads of information, a lot of guidance as well, and she made me feel quite sort of happy and content by always making sure, if I needed to call her or anything like that, she was always there…*’ (Guell et al. 2018, p. 1322). These positive experiences show the value of structured, non‐judgemental infant feeding support to mothers.

#### 3.5.2 Withheld or Conflicting Healthcare Professional Support

Women in all studies reported on the importance of HCPs when discussing their experience of formula feeding support. Mothers commonly reported the perception that HCPs intentionally withheld support for formula feeding, leading to a sense of neglect and feelings of marginalisation.

It was kind of like – ‘Well, you know formula feeding is bad? We'll not bother telling you anything about that’, and just push that to the side…it was almost like the midwives are not allowed to say anything.

(Lagan et al. 2014, p. e52)

Mothers perceived both antenatal and postnatal support for formula feeding negatively. They expected HCPs to provide advice and guidance on formula feeding, however some mothers indicated this was not the case, for example one woman described her antenatal classes as ‘*Very… pro breastfeeding… it was all like you know benefits of breastfeeding and we didn't really discuss formula or bottle‐feeding at all’.* (Lee 2007a, p. 306)

Mothers regularly reported that HCPs promoted breastfeeding and discouraged formula feeding, believing these to be the underlying motives shaping HCPs behaviours in delivering support. This led some women to believe that HCPs were not allowed to provide formula feeding support:

As soon as you go into hospital it's like breastfeeding‐breastfeeding, there's no advice and especially because of the Baby Friendly Initiative… They just don't provide you with the support.

(Guell et al. 2018)

Women expressed dissatisfaction with support they received from HCPs, which undermined their feeding decisions and strained the professional relationship. Some mothers expressed anger and resentment directly at HCPs and the healthcare system more broadly: ‘*I don't like the way that like midwives go on at you… they don't know the individual person and it's the individual person that it's about’.* (Lee 2007a, p. 304)

A major factor was conflicting and confusing advice from HCPs with the inconsistency in advice between professionals creating further uncertainty for mothers:

What I got was conflicting information between the health visitor, a midwife and my GP, because I found it really hard to work out do I stick to the tin… ‘cos that's what my midwife told me to do, … and then the health visitor comes round no, no, no, you should feed on demand’ cos he's crying he's hungry.

(Guell et al. 2018, p. 1323)

Contradictory advice left mothers with unanswered questions, creating further confusion: ‘*I asked the health visitor and she just said, “Oh keep him on stage one, that's fine.” But then I'm like, that's all well and good but why is there then a stage two? She didn't explain’.* (Conway et al. 2023, p. 108)

Lack of formal, professional support from HCPs, reinforced the unmet expectations of formula feeding mothers compared to breastfeeding mothers. This left mothers who were formula feeding feeling overlooked and unsupported:

I think for, the thing that really frustrates me is that everyone goes on about “breast is best” and then when you bottle‐feed they make you feel a bit like you've let your baby down, a bit like you're a failure, and there's all these like support groups, breastfeeding support groups in the community,… and there's not that for bottle‐feeding…

(Guell et al. 2018, p. 1323)

#### 3.5.3 Emotional Health and Wellbeing Impact on Mothers

Most studies highlighted the negative impact of inadequate professional infant feeding support on mothers' mental health. Mothers shared emotional reactions including defiance, anxiety, feelings of loneliness and isolation. Stigma surrounding formula feeding was commonly reported. Mothers often described feelings of inferiority compared to their breastfeeding peers, influenced by persistent pressure to repeatedly justify their feeding choices, and further reinforced by HCP behaviours.

They make you feel there is something wrong with you… you are made to feel if you don't do it, you are doing your child a mis‐justice.

(Thomson et al. 2015, p41)

These types of experiences resulted in feelings of shame, inadequacy and in some cases, behaviour characterised by deception or guilt, with women concealing their formula feeding to avoid criticism: ‘*I used to hide it’* (Lee 2007a, p. 304)

You're made to feel as if it's like smoking around a child… A lot of feeding (goes) underground.

(Lakshman et al. 2011, p. 678)

Women described the postpartum period as a vulnerable time, characterised by physical exhaustion and emotional fragility. Many described waiting for HCPs to offer proactive help, which sometimes never materialised, or feeling unsupported and pressured to breastfeed. Emotional vulnerability, combined with a lack of formula feeding support, intensified feelings of fear and shame:

I felt so guilty and bad about giving up, but I just couldn't stand the pain. When I was in hospital I had to go and get my own bottles and make them up. I […] felt really frowned upon, and made to feel really bad. I was really frightened of saying “I don't want to.” I was in fear of telling the midwife.

(Thomson et al. 2015, p. 40)

## 4 Discussion

This qualitative systematic review aimed to identify and synthesise research into mothers' experiences of formula feeding support in the UK and Ireland. Ten research papers were identified from which data were synthesised. Nine descriptive themes were distributed across three analytical themes.

Overall, the review found that mothers perceived a lack of support with formula feeding their babies, sometimes leading parents to rely on informal or commercial sources of information about infant feeding. This feeling of a lack of support is not unique to women who formula feed and exists in a broader context of stretched maternity services (Department of Health and Social Care, NHS England and The Rt Hon Wes Streeting 2025). A 2017 survey of women's experiences of maternity services identified baby feeding as the greatest area of unmet need for support (The National Federation of Women's Institutes NFWI and NCT 2017). A lack of support for breastfeeding is directly related to an increased need for formula feeding and it is recognised that infant feeding decisions lie within a complex system of influences including health services, family and social networks and the wider community infrastructure (Thomson et al. 2022). To holistically support infant feeding, improving support for breastfeeding should be a priority, with action required across government, healthcare, and society (Pérez‐Escamilla et al. 2023). Recognising the intersection of formula feeding and socioeconomic disadvantage (McAndrew et al. 2012) and the associated structural inequalities in care, action to holistically support infant feeding would contribute to the reduction in health inequalities. In this review, women who had intended to breastfeed often felt they lacked knowledge and confidence to formula feed, and reported feeling underprepared, confused, and uncertain about safe formula feeding practice.

For the women included in this review, formula feeding support was often felt to be missing, minimal, conflicting, or inconsistent. Although women did not express any concerns about the impact of the lack of support on infant health, they did report a harmful impact on their own emotional health and wellbeing, expressing a variety of negative emotional reactions such as anger, defiance, and frustration. Mothers felt anxious due to the information gap, with the lack of support influencing feelings of isolation and loneliness in their identity as a formula‐feeding mother. This highlights the importance of consistent, non‐judgemental support emphasised in national guidance (National Institute for Health and Care Excellence 2021; UNICEF 2025) Women sometimes struggled with their maternal identity due to the stigma of not breastfeeding and perceptions of ‘not doing their best’. Similarly, the social and cultural expectations to breastfeed heightened the feeling of inadequacy. These findings echo the review into research on the social norms of motherhood (Schmidt et al. 2023) where mothers were reported to experience feelings of failure and confusion when they make the decision not to breastfeed.

The findings in this review resonate with the 2009 systematic review of the international quantitative and qualitative literature on parents' experiences of bottle‐feeding (Lakshman et al. 2009), which reported that women do not receive sufficient information and support from HCPs and experience negative emotions such as guilt, anger, uncertainty and a sense of failure. A survey study by Fallon et al. (2017) of mothers mainly resident in the UK reported that a high percentage of women who formula fed experienced negative emotions including guilt (67%), stigma (68%), and the need to defend their decision to use formula (76%), with most women reporting feeling unsupported by HCPs. The authors described this as a widespread public health issue requiring urgent attention from policy makers to protect the emotional well‐being of mothers who formula feed and a need to address formula feeding in a more woman‐centred manner.

This review highlights women's perceptions that, despite HCPs being identified as one of the primary sources for information and advice on infant feeding, they were unable or reluctant to support formula feeding. These findings contrast with current UK policy regarding the provision of postnatal advice which states that all parents should receive evidence‐based, non‐judgemental support for their chosen feeding method (National Institute for Health and Care Excellence 2021), although only two of the included studies were published since this date. The findings may reflect organisational or training constraints, for example limited time for postnatal contacts with women, or uncertainty among HCPs about how to support formula feeding within current standards. This review emphasises women's perceptions of a lack of support for formula feeding compared to breastfeeding support. Similarly, a 2025 review of UK peer support and community interventions for infant feeding found that most interventions were focused on breastfeeding, with limited consideration of other feeding approaches. Stakeholders felt that work is required to understand how women may be supported to safely bottle‐feed and mixed feed in a non‐stigmatising manner, and the potential for inequities that might arise when seeking support for these approaches (Evans et al. 2025).

Within this review, positive experiences of a woman‐centred approach to supporting infant feeding were reported in one intervention trial of healthier formula feeding which addressed amounts of formula, infant satiety cues and recognising that crying was not always due to hunger (Guell et al. 2018) and one study evaluating a programme for a planned trial (Lakshman et al. 2011). Positive experiences of woman‐centred peer support for formula feeding were also reported by women taking part in the ABA‐feed trial (Clarke et al. 2025). These positive intervention experiences indicate the acceptability of additional woman‐centred support for women who formula feed. This support would align with the UK BFI standards of non‐judgemental, compassionate, parent‐centred support which should be provided regardless of infant feeding methods (UNICEF 2025).

### 4.1 Implications for Research

Further research is needed to understand HCP perspectives on providing formula feeding support and their awareness of guidance and how to operationalise it. Training needs to be implemented that enables HCPs to know what they can say to women about formula feeding and how to say it, while aligning with Baby Friendly principles and NICE guidance and addressing women's informational needs.

### 4.2 Strengths and Limitations

This review aimed to synthesise qualitative evidence on mothers' experiences of formula feeding support in the UK and Ireland. However, as no studies were found from Ireland, insights were limited to the UK. We acknowledge the potential for variation in support and experiences of support across UK healthcare settings.

The review excluded unpublished literature (e.g., theses and grey literature) which could had led to publication bias.

Selection criteria for this review included studies from 1992 onwards, with only two of the included studies published post‐2020. It is possible that women's experiences of formula feeding support have changed over time.

A methodologically robust approach was taken to undertaking the review using ENTREQ guidance. The quality of the underpinning evidence was good, although studies often had poor descriptions of relationships between the researcher and participants.

Only half of the included studies solely recruited women who were exclusively formula feeding. This could influence the findings due to the possible different experiences of support based on feeding intention and route to formula feeding.

## 5 Conclusion

This review provides insights into mothers' experiences of formula feeding support in the UK, highlighting the absence of information specifically from formal sources such as HCPs and the negative impact this has on mothers' emotional wellbeing. Although this review focused on support in its broadest sense, HCPs were a key focal point for mothers with a conflict between expectation and reality. It is not only important to provide parents with information, advice, and support, but necessary to enable safe formula feeding practice and maternal wellbeing.

## Author Contributions

J.C. and K.J. initiated and conceptualised the review. Review questions and design were developed by E.J., J.C. and K.J. E.J. and G.H. conducted the literature searches, with guidance from J.C. and K.J. E.J., D.L., G.H. and J.C. screened title and abstracts and full text. E.J. and D.L. carried out data extraction and analysis with support of J.C. and K.J. E.J., J.C. and G.H. prepared the initial draft with critical input of D.L. and K.J. All authors read and approved the final version of the manuscript.

## Conflicts of Interest

JC and KJ were investigators on the ABA‐feed study [NIHR129182] which included support for formula feeding. EJ, DL and GH report no conflicts of interest.

## Supporting information

Supp file ‐ Search strategy ‐ mothers' experiences of FF support.

## Data Availability

The data that support the findings of this study are available from the corresponding author upon reasonable request.
