# Supplementary material for: Mothers' Experiences of Formula Feeding Support in the UK: A Qualitative Systematic Review
Source: Matern Child Nutr. 2026 Apr 17;22(2):e70182. doi: 10.1111/mcn.70182 (PMC13087542; doi:10.1111/mcn.70182)
Supplement: Supplementary file 1 — Supp file ‐ Search strategy ‐ mothers' experiences of FF support. [file MCN-22-e70182-s001.docx]

**Supplementary file: search strategy for all databases**

|  | **CINAHL** |
| --- | --- |
| S1 | (MH “Mothers+*) |
| S2 | Mother* |
| S3 | (MH “Infant Feeding, Supplemental”) |
| S4 | (MH “Bottle Feeding”) |
| S5 | (formula or bottle) N2 feed* |
| S6 | Support or advi* or assist* or guid* |
| S7 | Experience or view or attitude or opinion or perception |
| S8 | S1 OR S2 |
| S9 | S3 OR S4 OR S5 |
| S10 | S7 AND S8 AND S9 |
| S11 | S6 AND S10 |
| S12 | Qualitativ* or interview or focus group |
| S13 | S11 AND S12 |

|  | **Medline** |
| --- | --- |
| 1. | exp Mother-Child Relations/ |
| 2. | mother*.tw. |
| 3. | exp Bottle Feeding/ |
| 4. | exp Infant Formula/ |
| 5. | ((formula or bottle or infant) adj2 feed*).tw. |
| 6. | (Experience* or view* or attitude* or opinion* or perception* or perceive* or satisf* or emotion* or feeling*).tw. |
| 7. | (Support* or advi* or assist* or guid* or educat* or "peer support" or intervention* or midwi?e* or "health visit*" or "general practitioner*" or GP* or doctor* or nurs* or "health profession*" or family or friend* or relative* or online or "social media").tw. |
| 8. | exp qualitative research/ |
| 9. | exp interview/ |
| 10. | exp Focus Groups/ |
| 11. | (qualitative or interview* or "focus group*").tw. |
| 12. | 1 or 2 |
| 13. | 3 or 4 or 5 |
| 14. | 12 and 13 |
| 15. | 6 and 7 |
| 16. | 14 and 15 |
| 17. | 8 or 9 or 10 or 11 |
| 18. | 16 and 17 |
| 19. | exp United Kingdom/ |
| 20. | (national health service* or nhs*).ti,ab,in. |
| 21. | (gb or "g.b." or britain* or british* or uk or "u.k." or united kingdom* or england* or english* or northern ireland* or northern irish* or scotland* or scottish* or wales or welsh* or ireland or irish*).ti,ab,in,jw,cp. |
| 22. | 19 or 20 or 21 |
| 23. | 18 and 22 |
| 24. | limit 23 to yr="1992 -Current" |

|  | **PsycINFO** |
| --- | --- |
| 1. | mother.mp. |
| 2. | exp mothers/ |
| 3. | exp Bottle Feeding/ |
| 4. | ((formula or bottle or infant) adj2 feed*).mp. [mp=title, abstract, heading word, table of contents, key concepts, original title, tests & measures, mesh word] |
| 5. | "formula milk".mp. |
| 6. | (Experience* or view* or attitude* or opinion* or perception* or perceive* or satisf* or emotion* or feeling*).mp. [mp=title, abstract, heading word, table of contents, key concepts, original title, tests & measures, mesh word] |
| 7. | (Support* or advi* or assist* or guid* or educat* or "peer support" or intervention* or midwi?e* or "health visit*" or "general practitioner*" or GP* or doctor* or nurs* or "health profession*" or family or friend* or relative* or online or "social media").mp. [mp=title, abstract, heading word, table of contents, key concepts, original title, tests & measures, mesh word] |
| 8. | exp qualitative methods/ |
| 9. | exp focus group interview/ |
| 10. | interview.mp. |
| 11. | exp focus group/ |
| 12. | focus group.mp. |
| 13. | 1 or 2 |
| 14. | 3 or 4 or 5 |
| 15. | 13 and 14 |
| 16. | 6 and 7 |
| 17. | 15 and 16 |
| 18. | 8 or 9 or 10 or 11 or 12 |
|  |  |
|  |  |
| 19. | 17 and 18 |
| 20. | (national health service* or nhs*).mp. [mp=title, abstract, heading word, table of contents, key concepts, original title, tests & measures, mesh word] |
| 21. | (gb or "g.b." or britain* or british* or uk or "u.k." or united kingdom* or england* or english* or northern ireland* or northern irish* or scotland* or scottish* or wales or welsh* or ireland or irish*).mp. [mp=title, abstract, heading word, table of contents, key concepts, original title, tests & measures, mesh word] |
| 22. | 20 or 21 |
| 23. | limit 19 to yr="1992 -Current" |
| 24. | 19 and 22 |

|  | **Web Of Science** |
| --- | --- |
| 1. | (gb or "g.b." or britain* or british* or uk or "u.k." or united kingdom* or england* or english* or northern ireland* or northern irish* or scotland* or scottish* or wales or welsh* or ireland or irish*) (Topic) |
| 2. | (national health service* or nhs*) (Topic) |
| 3. | #1 OR #2 |
| 4. | Mother* (Topic) |
| 5. | TS=((formula or bottle) near/2 feed* ) |
| 6. | (formula or infant) near/2 milk (Topic) |
| 7. | #5 OR #6 |
| 8. | Qualitative* or interview or “focus group” (Topic) |
| 9. | #4 AND #7 AND #8 |
| 10. | TS=(Support OR advi* OR assist* OR guid* OR educat* OR "peer support" OR intervention OR midwife* OR "health visit*" OR "general practitioner*" OR GP OR doctor* OR nurs* OR "health profession*" OR family OR friend* OR relative* OR online OR "social media") |
| 11. | TS=(Experience* OR view* OR attitude* OR opinion* OR perception* OR perceive* OR satisf* OR emotion* OR feeling*) |
| 12. | #10 OR #11 |
| 13. | #9 AND #12 |
| 14. | #3 AND #13 |

|  | **ASSIA** |
| --- | --- |
| 1. | noft(mother) |
| 2. | (noft(formula) OR noft(bottle)) NEAR/2 noft(feed*) |
| 3. | noft(Support) OR noft(advi*) OR noft(assist*) OR noft(guid*) OR noft(educat*) OR noft("peer support") OR noft(intervention*) OR noft(midwife*) OR (noft("health visitor") OR noft("health visitors")) OR noft("health visiting") OR (noft("general practitioner") OR noft("general practitioners")) OR noft(GP*) OR noft(doctor*) OR noft(nurse*) OR (noft("health profession") OR noft("health professional") OR noft("health professionals") OR noft("health professions")) OR noft(family) OR noft(friend*) OR noft(relative* online) OR noft("social media") |
| 4. | (noft(qualitative*) OR noft(interview) OR noft("focus group")) |
| 5. | noft(Experience*) OR noft(view*) OR noft(attitude*) OR noft(opinion*) OR noft(perception*) OR noft(perceive*) OR noft(satisf*) OR noft(emotion*) OR noft(feeling*) |
| 6. | noft(gb) OR noft("g.b.") OR noft(britain*) OR noft(british*) OR noft(uk) OR noft("u.k.") OR noft(united kingdom*) OR noft(england*) OR noft(english*) OR noft(northern ireland*) OR noft(northern irish*) OR noft(scotland*) OR noft(scottish*) OR noft(wales) OR noft(welsh*) OR noft(ireland) OR noft(irish*) |
| 7. | #1 AND #2 AND #4 AND #6 |
| 8. | #3 OR #5 |
| 9. | #7 AND 8 |
